# Estimating Vehicle and Pedestrian Activity from Town and City Traffic Cameras †

**DOI:** 10.3390/s21134564

**Published:** 2021-07-03

**Authors:** Li Chen, Ian Grimstead, Daniel Bell, Joni Karanka, Laura Dimond, Philip James, Luke Smith, Alistair Edwardes

**Affiliations:** 1 Data Science Campus, Office for National Statistics, Newport, South Wales NP10 8XG, UK; ian.grimstead@ons.gov.uk (I.G.); alistair.edwardes@defra.gov.uk (A.E.); 2 Urban Observatory, Newcastle University, Newcastle upon Tyne NE1 7RU, UK; daniel.bell2@newcastle.ac.uk (D.B.); philip.james@newcastle.ac.uk (P.J.); luke.smith@newcastle.ac.uk (L.S.); 3 Economic Statistics Hub, Methodology, Office for National Statistics, Newport, South Wales NP10 8XG, UK; joni.karanka@ons.gov.uk (J.K.); laura.dimond@ons.gov.uk (L.D.)

**Keywords:** traffic camera, deep learning, cloud computing, mobility, time series

## Abstract

Traffic cameras are a widely available source of open data that offer tremendous value to public authorities by providing real-time statistics to understand and monitor the activity levels of local populations and their responses to policy interventions such as those seen during the COrona VIrus Disease 2019 (COVID-19) pandemic. This paper presents an end-to-end solution based on the Google Cloud Platform with scalable processing capability to deal with large volumes of traffic camera data across the UK in a cost-efficient manner. It describes a deep learning pipeline to detect pedestrians and vehicles and to generate mobility statistics from these. It includes novel methods for data cleaning and post-processing using a Structure SImilarity Measure (SSIM)-based static mask that improves reliability and accuracy in classifying people and vehicles from traffic camera images. The solution resulted in statistics describing trends in the ‘busyness’ of various towns and cities in the UK. We validated time series against Automatic Number Plate Recognition (ANPR) cameras across North East England, showing a close correlation between our statistical output and the ANPR source. Trends were also favorably compared against traffic flow statistics from the UK’s Department of Transport. The results of this work have been adopted as an experimental faster indicator of the impact of COVID-19 on the UK economy and society by the Office for National Statistics (ONS).

## 1. Introduction

Today’s traffic monitoring systems are highly integrated within urban infrastructures, providing near-ubiquitous coverage in the busiest areas for managing flows, maintaining safety, and performance analysis. Traffic cameras and Closed-Circuit TeleVision (CCTV) networks comprise much of these systems, supplying real-time information primarily intended for use by control room operators.

A major advantage of CCTV networks is their scalability. A monitoring system can range from a single camera to potentially thousands of cameras across a city or region accessible from a unified network. The imagery these cameras provide can be a further source of data when combined with machine learning and object detection. Through these, it is possible to obtain data for the modeling of traffic and pedestrian flows, as part of micro- and macro-simulation systems, ranging from the discrete movements and interactions of a single entity to overall flow characteristics on a regional scale by simultaneously analyzing conditions across multiple camera sensors, respectively [1,2,3,4]. Recent years have seen considerable research focus on improved machine learning for CCTV applications, and a range of open-source, research, and commercial implementations are now widespread.

A major concern for public policy responding to the COVID-19 pandemic has been to quantify the efficacy of interventions, such as social distancing and travel restrictions, and to estimate the consequences of changes in travel patterns for the economy and spread of the virus. The UK has thousands of publicly accessible traffic cameras with providers ranging from national agencies such as Highways England, who stream images from 1500 cameras located across the major road system of England, and Traffic Wales; to more localized authorities such as Transport for London, with more than 900 cameras, and Reading Borough Council.

There are a number of strengths to traffic cameras as a source of mobility data:

- The data are very timely. Cameras may provide continuous video, or stills updated many times each hour, and these images can be accessed almost immediately following capture.
- A wide range of different types of moving objects can be determined, including cars, buses, motorcycles, vans, cyclists, and pedestrians, all of which can provide different useful information from changes in individual behavior to impacts on services and local economies.
- The source is open. Data are widely accessible to groups with differing interests and reusing a public resource adds value to public investment without incurring additional collection cost.
- Cameras provide coverage over a range of geographic settings: the center of towns and cities, as well as many areas that show either retail or commuting traffic and pedestrian flows.
- There is a large network of traffic cameras capturing data simultaneously, allowing the selection of individual cameras for different purposes whilst still retaining acceptable coverage and precision.
- Individuals and vehicles are, in almost all cases, not personally identifiable, e.g., number plates and faces, due to the low resolution of the images.

However, the source is also not without data quality problems. In order to generate useful mobility statistics, objects of interest within images need to be automatically detected. These exhibit wide variabilities due to the different styles of vehicles and their sizes in the image, varying by distance from the camera. The quality of individual images can vary substantially due to weather, time of day, camera setting, and image resolution and quality. These factors make robust detection problematic. The images are generated frequently, creating large data volumes, and are dispersed across multiple sites of different camera operators. This complicates their collation and processing into statistics. The geographic coverage of cameras is also biased according to the desire to show traffic flow at strategic sites and the open data policies of different camera operators in different locations.

In this paper, we investigated these issues within the context of a National Statistical Office producing early experimental data to monitor the impact of COVID-19 on the UK economy and society [5]. In Section 2, we describe the source of data used and methods for selecting cameras pertinent to meet our objectives. The system architecture is described in Section 3, comprising a scalable pipeline in the Cloud to process these data sources. This is followed by the description of a deep learning-based machine learning pipeline in Section 4, which allows the detection of pedestrians and vehicles from traffic camera images as a way of measuring the busyness of different towns and cities in the UK. Here, we discuss the comparative analysis on the cost, speed, and accuracy of different machine learning methods to achieve this, and present pre- and post-processing methods to improve the accuracy of results. Section 5 introduces the statistical processing of imputation and seasonal adjustment on the raw time series, and then the raw and processed time series are validated with other sources from ANPR and traffic activities from the Department for Transport (DfT) in Section 6. Discussion and conclusions are presented in Section 7 and Section 8, respectively, with plans for further work highlighted. This work has been made available for public authorities and researchers to re-deploy on an open basis.

### Background

Extracting information from traffic cameras requires semantic entities to be recognized in the images and videos they capture. This is a popular topic in computer vision, particularly the field of object detection [6,7,8,9,10,11]. Deep learning research has achieved great success in this task through the development of algorithms such as the You Only Look Once (YOLO) series [12] and Faster-Region-based Convolutional Neural Network (Faster-RCNN) [13,14].

Traditional computer vision machine learning techniques have been employed by authors such as Wen et al. [15]. Here, Haar-like features and AdaBoost classifiers are used to achieve accurate vehicle detection. Such techniques were also enhanced to make use of a vehicle indexing and search system [16]. Another well-established technique for accurate vehicle detection takes the combined approach of background extraction, foreground segmentation, and blob classification [17,18]. For even further performance and reliability, Haar-features, histograms of Oriented Gradients (HOG), and blob classification can be combined [19]. Techniques prior to 2011 can be found in a detailed review by [6].

A major benefit of deep learning-based methods over traditional detection techniques is robust performance within challenging monitoring conditions. This compensates for poor lighting conditions [20], or the substandard camera resolution [7,21] used in the YOLOv2 deep learning framework to recognize multiple vehicle categories. In [22], Mask-RCNN was utilized, an extension of Faster-RCNN, to define precise segmentation masks of vehicles within traffic footage. Similarly, authors Hua et al. [23], Tran et al. [24], and Huang [25] all made use of the Faster-RCNN variant, employing resnet101 for the feature extractor. In addition to these, Fan et al. [26] demonstrated the use of Faster-RCNN from a vehicle platform, utilizing the KITTI Benchmark dataset.

Researchers in the UK have applied these models to traffic cameras to see if the data can be used to improve decision-making in COVID-19 policies. The Alan Turing Institute worked with the Greater London Authority (GLA) to assess activity in London before and after the lockdown [27]. Their work is, in part, based on the air pollution estimation project of University College London (UCL) [28], which detects and counts vehicles from JamCam (London) traffic cameras to estimate air pollution using a deep learning model based on YOLOv3. Newcastle University’s Urban Observatory [29] has worked on traffic cameras and Automatic Number Plate Recognition (ANPR) to measure traffic and pedestrians in North East England using the Faster-RCNN model [30,31]. Similarly, the Urban Big Data Centre at the University of Glasgow has explored using the spare CCTV capability to monitor activity level during the COVID-19 pandemic on streets in Glasgow [32,33]. A few companies have also undertaken related work using their own networks of cameras. Vivacity Labs [34] demonstrated the use of their commercial cameras and modeling to assess compliance with social distancing. Meanwhile, camera-derived data on retail footfall from Springboard [35] have also been used in ONS as part of experimental data [5] looking at the economic impacts of COVID-19. Further afield, researchers at the University of Cantabria, Spain, have analyzed the impact of COVID-19 on urban mobility by comparing vehicle and pedestrian flow data (pre- and post-lockdown restrictions) from traffic control cameras [36].

Despite not employing traffic cameras in their research, Zambrano-Martinez et al. [37] presented a useful approach in modeling an urban environment based upon busyness metrics such as vehicle loads and journey times. Likewise, Costa et al. [38] did not focus on utilizing existing traffic camera networks, but rather outlined a concept by which existing telecommunication infrastructure can be leveraged to achieve accurate traffic flow modeling and prediction.

There are a number of unresolved issues in realizing the use of open traffic camera imagery for real-world insight. Most notably, there has been substandard sets of quality training data and models when compared against similar computer vision applications within other domains. The versatility of these tools and resources are of critical consideration for scalable applications (where internal and external camera conditions are variable), and for monocular camera setups (where accurate 3D geometry extraction is often either impossible or impractical). Since 2017, the AI City Challenge has gathered researchers from around the globe to address these problems, as well as to solve the complex issues that limit accurate data retrieval within real-world contexts [39]. With each challenge, increasing focus has been given to analyzing multi-camera networks that simulate existing large-scale urban systems. For example, in 2020, participants were encouraged to compete in one or more of the following four categories: Multi-Class Multi-Movement Vehicle Counting, City-Scale Multi-Camera Vehicle Re-Identification, City-Scale Multi-Camera Vehicle Tracking, and Traffic Anomaly Detection [40]. Among successful experiments, object detection models from the YOLO series and Faster-RCNN were utilized for their superior speeds and performance on consumer-grade GPUs. Similarly, challenges have been organized by UA-DETRAC for the AVSS conference 2019, Taipei, Taiwan, whereby participants contributed innovative solutions for improving computer vision vehicle detection and counting performance [41]. Here, the UA-DETRAC Benchmark Suite for multi-object detection and tracking was used exclusively.

The development of benchmark standards is integral for measuring the relative performance of deep learning-aided traffic monitoring applications. Notable benchmarks include the CityFlow Benchmark dataset for multi-camera, multi-target vehicle reidentification, which contains almost 3.5 h of 960p footage, of traffic at highways and intersections within the US [42], and UA-DETRAC Benchmark Suite containing 10 h of footage extracted from 24 locations in Beijing and Tianjin, China [41].

As with traffic monitoring applications, several notable pedestrian detection and tracking benchmarks have been developed. The CrowdHuman benchmark specializes in pedestrian detection within densely populated and highly diverse scenes, making for many occlusion instances [43]. In total, the dataset contains 470,000 identified persons across 24,000 images, for training, testing, and validation purposes. The Multiple Object Tracking (MOT) benchmark extends upon basic detection principles by providing evaluation datasets for multi-target pedestrian tracking [44]. Data are crowdsourced and distributed periodically with each new benchmark challenge. Built upon the CityScapes dataset, the CityPersons benchmark dataset consists of 19,654 unique persons captured across 18 central-European cities [45,46]. Footage was recorded from a vehicle platform under varying weather conditions. The Caltech Pedestrian Detection Benchmark consists of 2300 labeled pedestrians [47]. Dataset footage has been captured from a road vehicle perspective during ‘regular’ Los Angeles metropolitan traffic conditions. The EuroCity Persons detection benchmark consists of 238,200 persons within 47,300 images from road vehicle perspectives [48]. Data have been collected from across 31 cities in Europe, in all seasonal weather and day/night lighting conditions.

One combined traffic and pedestrian monitoring benchmark is KITTI [49]. This dataset was captured using an array of vehicle platform sensors, including a stereo camera rig (for computer vision dataset), as well as a Velodyne Laserscanner and Global Positioning System (GPS) (for spatial and temporal ground truth validations). From these, 3D object detection and tracking could be achieved. In total, the dataset consists of over 200,000 annotations, in some instances with up to 30 pedestrians and 15 cars per image, in urban and rural environments alike from the city of Karlsruhe, Germany.

Previous work left a research gap with a lack of publicly available low-resolution traffic camera imagery and test data sets, so we provide a publicly available dataset of 10,000 traffic images [50] and also aim to release 4000 annotated images.

## 2. Data

**Figure 1:**
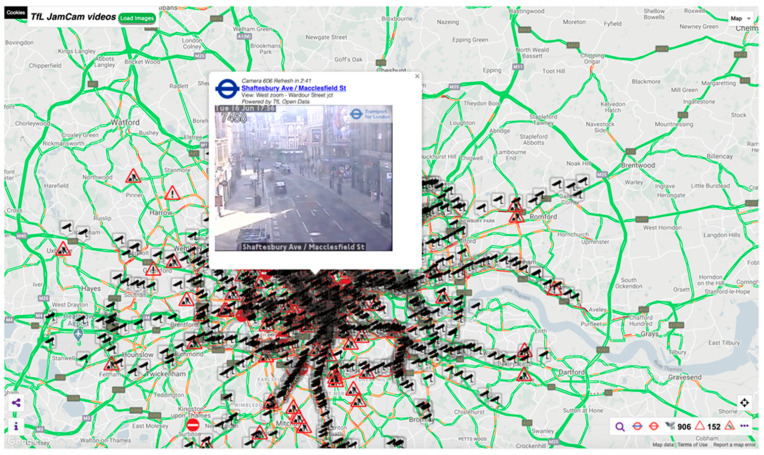
Example of a website providing access to traffic camera data streams provided by the local government body Transport for London via their JamCam open data service (screenshot from https://www.tfljamcams.net (accessed on 28 June 2021) data © 2021 Google; Contains OS data © Crown copyright and database rights 2021 and Geomni UK Map data © and database rights 2021).

The UK has thousands of traffic cameras, particularly in major cities and towns; for example, in London alone, there are nearly 1000 accessible cameras (see Figure 1 from the JamCam website). These data are available in the public domain where automatic periodic static images are updated from traffic camera networks across the UK.

Appendix A lists open websites we have reviewed where camera images are publicly available. We selected a subset of locations from that list and built up a profile of historical activity from their cameras. Table 1 lists the sources we selected.

These locations give broad coverage across the UK whilst also representing a range of different sized settlements in both urban and rural settings.

### 2.1. Camera Selection

**Figure 2:**
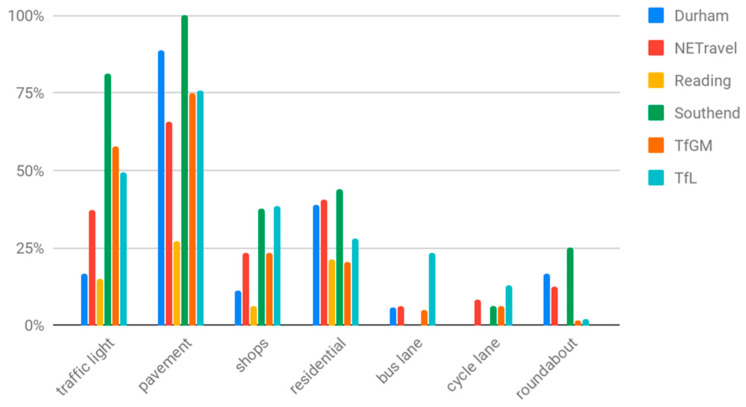
Percentage of cameras, for each source of open traffic camera images, that include the given scene element category within their frame of view.

In this research, we were interested in measuring changes in the general busyness of places, rather than monitoring traffic flow per se. Not all cameras are useful for measuring this; many of the cameras are focused on major trunk roads or located on country roads without pavements. To select cameras that were representative of the busyness of local populations, we manually tagged cameras if they contained certain scene elements, e.g., pavements and cycle lanes, within the scenes they captured. The elements we tagged are shown in Figure 2.

We scored cameras based on their mix of these scene elements to determine which were more likely to represent ‘busyness,’ such as street scenes with high footfall potential. The higher the total score, the more likely the cameras were expected to include both pedestrians and vehicles. The scores, determined empirically, were given in Table 2.

**Figure 3:**
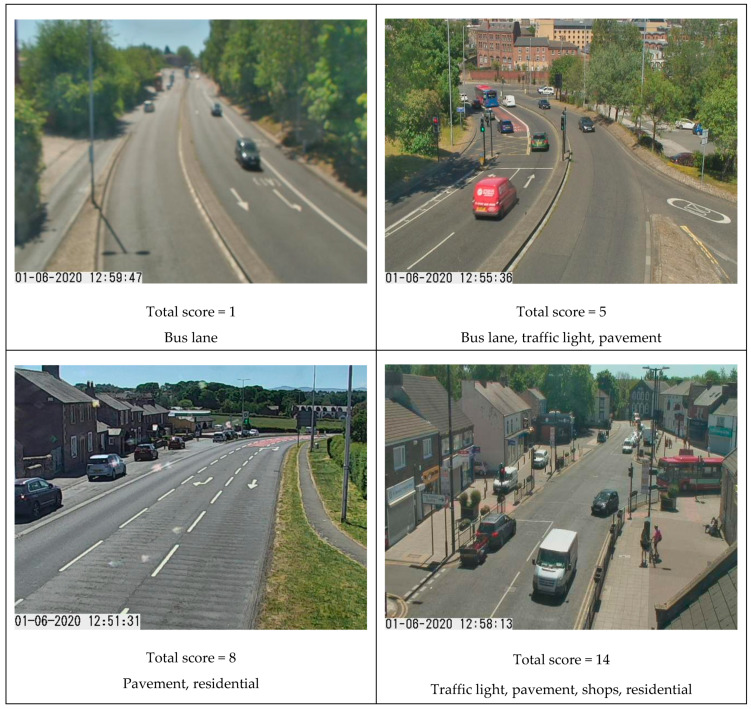
Selection of sample images from cameras with differing total ‘busyness’ scores (images captured 13:00, 1 June 2020) obtained from Tyne and Wear UTMC Open Data Service under Open Government Licence v3.0.

We used the total scores to decide if a camera was used; example images with total scores from 1 to 14 are presented in Figure 3, noting the increasing likelihood of pedestrians in the image as coverage area becomes more residential or with shops.

We examined the distribution of scores amongst image providers, with total scores of eight or above noted as having an even distribution amongst providers, and likely to contain shops or residential areas (a source of pedestrians). A threshold value of eight was chosen to represent a reasonable likelihood of capturing footfall whilst reducing sample size (such as excluding areas without pavements).

These types of scenes showed a reasonable spread for all image providers, meaning the different geographic areas covered by providers have a reasonably consistent distribution of street scenes. To avoid one area overwhelming the contribution from another, we also opted to keep each geographic area as a separate dataset. We therefore investigated the time series behavior for each area independently, rather than producing an aggregate time series for the UK. How to produce such an output using post-stratification is being considered as part of future work.

### 2.2. Annotation of Images

**Figure 4:**
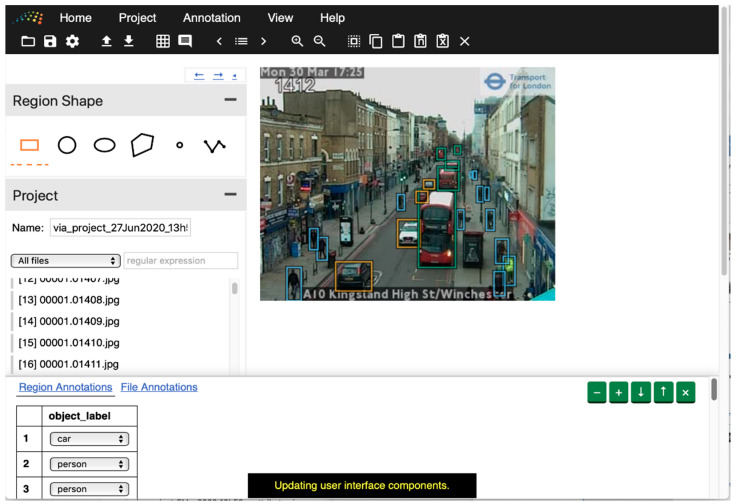
Example of the VGG Image Annotator tool being used to label objects on an image of a London street (TfL open data): orange box: car, blue box: person, green box: bus.

Annotated data are required to train and evaluate models. For this research, we created a dataset of manually labeled images. We used an instance [51] of the VGG Image Annotator (VIA) [52] application to label our data, hosted courtesy of the UN Global Platform [53]. We labeled seven categories: car, van, truck, bus, person, cyclist, and motorcyclist. Figure 4 shows an interface of the image annotation application.

Two groups of volunteers from the Office for National Statistics (ONS) helped label approximately 4000 images from London and North East England. One group labeled a randomly selected set of images without consideration of the time of day; the other labeled randomly selected images but with an assigned time of day. Random selection helped to minimize the effect of bias on training and evaluation. However, we also wanted to evaluate the impact of different illumination levels and so purposely stratified sampling by fixed time periods for some images.

Table 3 presents counts on the labeled data. The Urban Observatory, Newcastle University provides a well-trained Faster-RCNN model based on 10,000 images from North East England traffic cameras, and the above datasets are thus used to evaluate the models.

## 3. Processing Pipeline: Architecture

A processing pipeline was developed to gather images and apply models to detect numbers of pedestrians and vehicles. This architecture can be mapped to a single machine or a cloud system; we opted to use Google Compute Platform (GCP), but other platforms such as Amazon’s AWS or Microsoft’s Azure would provide relatively equivalent services.

**Figure 5:**
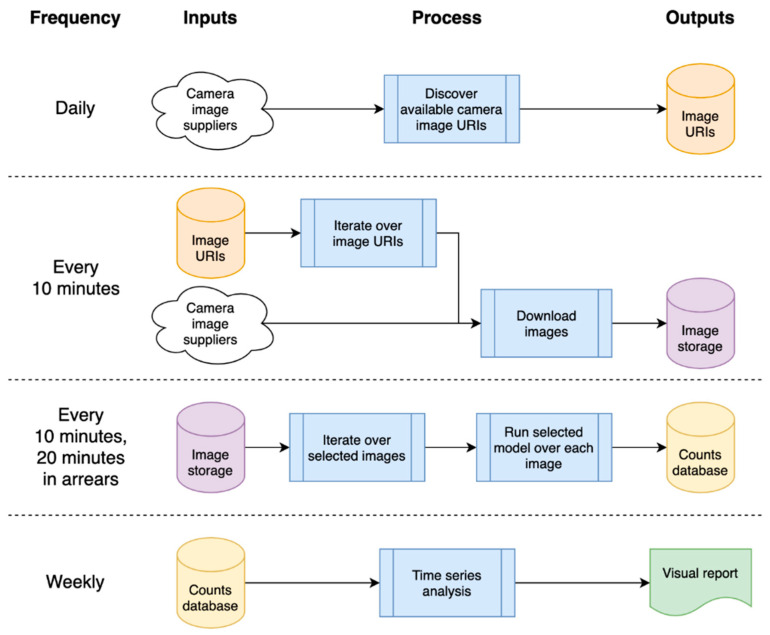
High-level architecture of processing pipeline, showing timings and input/output dependencies.

The solution pipeline was developed in the Cloud, as this removed the need for machine maintenance, and provided capacity for large (managed) data storage and on-demand scalable compute. Remote, distributed working is also supported, which was extremely useful as we developed this during the pandemic and, hence, were all working remotely. Code in the pipeline was run in ‘bursts’; as cloud computing is only charged when used, this was a potential saving in compute costs. Our overall architecture is presented in Figure 5, where blue boxes indicate a process and other colors represent storage, the color being used to remind the user that this is the same object being referred to as a dependency.

The system depends on a list of available camera image Uniform Resource Locator (URL)s, which are refreshed daily; these camera images are then downloaded every 10 min, and analyzed 20 min later (as we need a before/after image for filtering—see later in Section 4.3 Static Model). The analysis generates object counts that are stored in a database, which is processed weekly to generate a time series and related statistical reports.

Google Cloud Functions [54] were used. These are stand-alone, stateless code that can be called repeatedly without causing corruption—a key consideration to increase the robustness of the functions. A major advantage occurs when we make multiple requests to a cloud function. If it is busy servicing another request and there is sufficient demand, another instance will be spun up automatically to take up the load. These instances are not specifically charged for, as charges are for seconds of compute used and memory allocated. Hence, running 100 instances to perform a task costs little more than running a single instance 100 times.

This on-demand scalability enabled the code for downloading images to take advantage of GCP’s internet bandwidth. Over a thousand images could be downloaded in under a minute and the system could easily scale to tens of thousands of images. To cope with a potentially enormous quantity of imagery, cloud storage buckets were used to hold the data—these are named file storage locations within GCP that are secure and allocated to the project. Processing the images through a model to detect cars and pedestrians resulted in counts of different types of detected objects being written into a database. We used a managed database, BigQuery [55], to reduce maintenance. The database was used to share data between the data collection and time series analysis, reducing coupling. The time series analysis was a compute-intensive process taking hours to run due to imputation overhead (described in Section 5). Processing was scheduled daily using cloud functions. The system is highly flexible; it is not restricted to images but is merely a large-scale data processing pipeline where data are fed into filters and then processed by models to eventually generate object counts. For instance, the input data could include audio files where we are looking to detect signals, or text reports where we wish to count key phrases or themes. It is also cost-efficient. The entire cloud infrastructure to produce weekly time series costs approximately GBP 10/day to run, processing ~800 cameras and, hence, ~115,000 images per day.

## 4. Deep Learning Model

**Figure 6:**
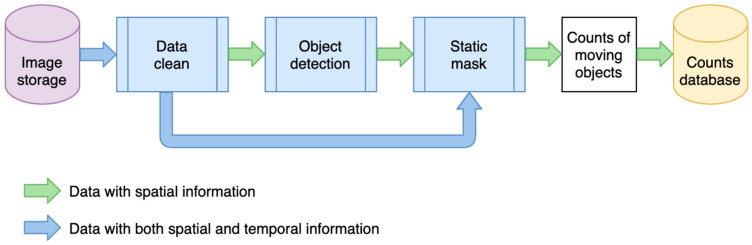
Data flow of the modeling process.

The deep learning pipeline consists of data cleaning, deep learning-based object detection, and a static filter (post-processing). Figure 6 describes the modeling process. The data flow shown in green processes spatial information from a single image. Spatial information refers to information from pixels and their neighbors within the two-dimensional space or a single image (rather than its location in geographic space). Here, each image is processed independently without considering other images captured before or after it. The blue workflow processes both spatial information, of individual images, and temporal information from their most recent neighboring images in time. The data cleaning step uses the temporal information to check if an image is duplicated, dummy, or faulty. As it may be possible to identify number plates or faces on some high-resolution images, the data cleaning also down-samples images from high resolution to low resolution to blur these details. The object detection step uses spatial information to identify semantic objects in a single image using a deep learning model. The static mask is a post-processing step that uses temporal information over a sequence of images to determine whether these objects are static or moving.

### 4.1. Data Cleaning

**Figure 7:**
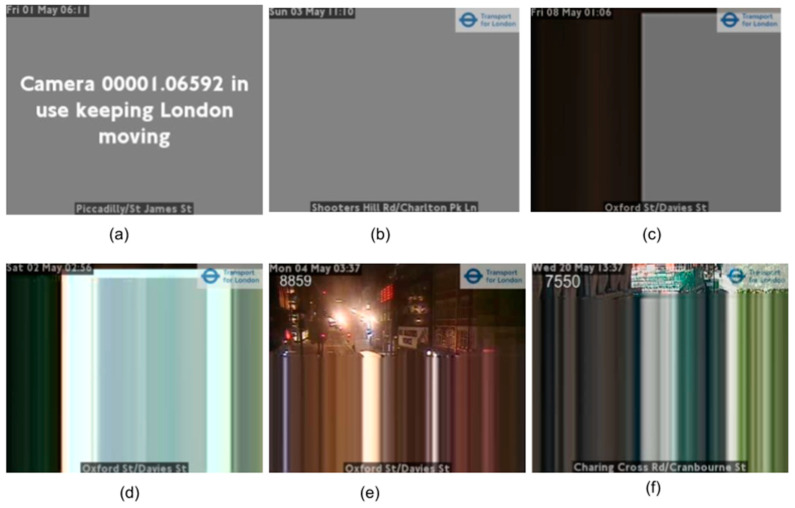
Examples of corrupt or problematic images. (**a**) Camera offline usually due to ongoing incident; (**b**,**c**) cameras offline; (**d**–**f**) examples of distorted images (images TfL Open Data).

A number of issues were found when using these low-quality camera images. In principle, new images are fetched every 10 min from cameras, for example, via an API or from cloud storage container. However, in practice, images can be duplicated if the hosts have not updated them in time. Cameras can be periodically offline, and when this happens, they display placeholder images. Images can also be partly or completely distorted due to physical problems with the cameras. Some examples of problematic images are shown in Figure 7. These types of images will impact counts, so data cleaning is an important preliminary step to improve the quality of statistical outputs.

By examining images from London and North East England traffic, two common characteristics of unusable images were identified: large continuous areas consisting of different levels of pure grey (R=G=B) (Figure 7a–c) and horizontal rows repeated in whole or part of the image (Figure 7d–f). To deal with the first issue, a subsample of the colors in the image was used to detect if a single color covered more than a defined threshold area. We found that sampling every 4th pixel both horizontally and vertically (1/16th of the image) gave sufficient coverage; further down-sampling pixels from 256 levels to 32 levels in each of the red, green, and blue was sensitive enough to correctly detect all nonphotograph images, such as ‘Camera 00001.06592 in use keeping London moving’ in Figure 7a, where a single color covered >33% of an image. For the partly or completed distorted images, a simple check to determine if consecutive rows contained a near-identical sequence of pixels (allowing for variation due to compression artefacts) was used. If a significant portion of the image contained such identical rows, we marked the image as ‘faulty’.

**Figure 8:**
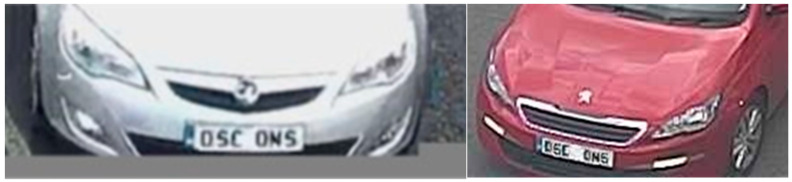
Modified examples of disclosive number plates captured on traffic cameras.

**Figure 9:**
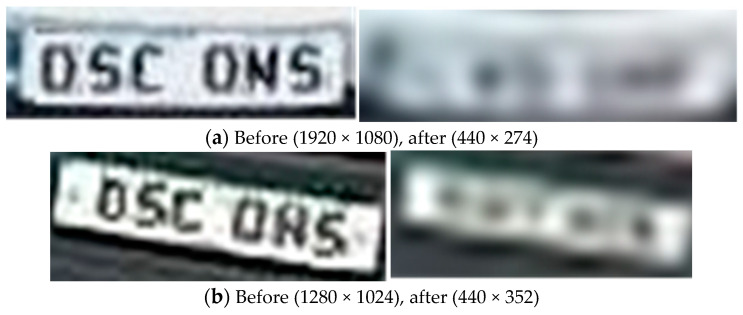
Examples of images before and after down-sampling.

Some local councils have deployed high-definition cameras, and this brings General Data Protection Regulation (GDPR) issues where number plates or faces are potentially identified. Figure 8 shows two modified examples of disclosive number plates captured on CCTV images. To assess the risk of data protection, we recorded a 24 h period of images from all the traffic cameras and manually examined the resolution and risk of recognizing number plates and faces. We observed that the most common image width of these datasets was 440 pixels, and no legible number plates were identified under this width. A width of 440 was thus chosen as our threshold to deem an image as being of ‘higher resolution’ if exceeded. The down-sampled height depends on the ratio of width before and after (440 pixels). Figure 9 shows some examples of images before and after down-sampling.

**Figure 10:**
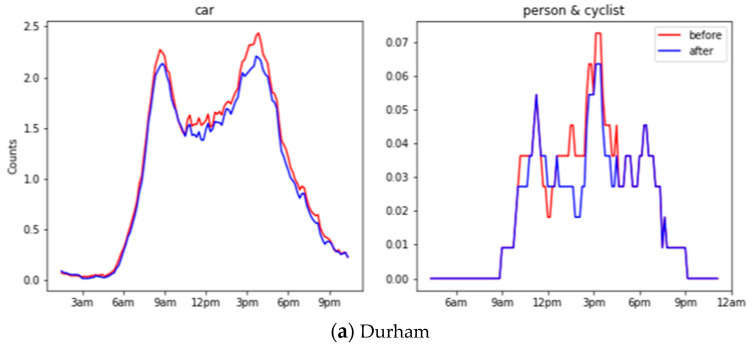

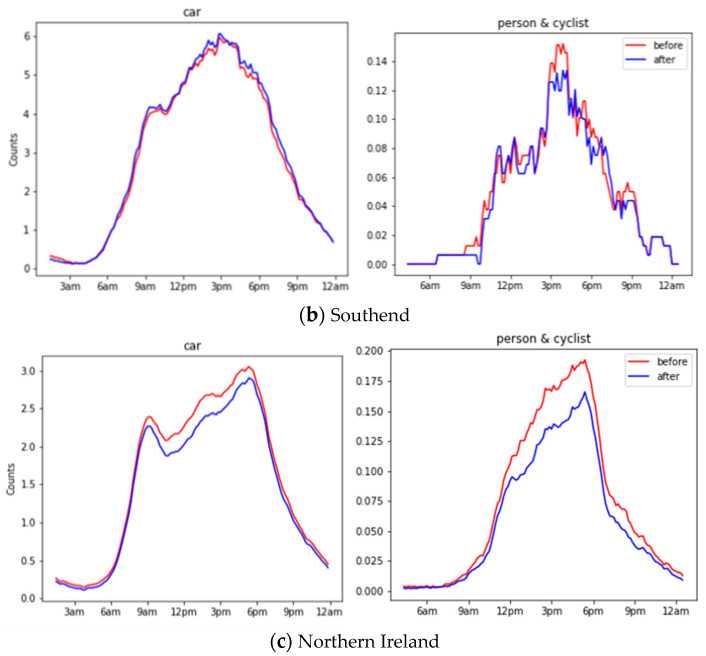
Object counts before and after down-sampling during 24 h for Durham, Southend and Northern Ireland.

We have evaluated the object counts generated before and after down-sampling across all locations on the 24 h recorded images. Figure 10 shows the object counts in different locations before and after down-sampling during 24 h. Traditional evaluation on object recognition requires a time-costing manual label, as mentioned in Section 2.2. Alternatively, we adopted the Pearson correlation to compare two time series, where 1 means perfectly correlated, 0 means no correlation, and a negative number means opposite correlated. A high Pearson correlation score reflects the trivial influence from the down-sampling operation. In general, cars have a 0.96 correlation, whereas a person and cyclist have a 0.86 correlation, and this shows a significant close correlation before and after down-sampling.

### 4.2. Object Detection

#### 4.2.1. Deep Learning Model Comparison

Object detection is used to locate and classify semantic objects within images. We compared a number of widely used models including the Google Vision API, Faster-RCNN, and the YOLO series. Google Vision API provides a pre-trained machine learning model to ‘assign labels to images and quickly classify them into millions of predefined categories’ [56]. It helps with rapidly building proof-of-concept deployments but is costly with slower speed and worse accuracy compared to the other methods. Faster-RCNN was first published in 2017 [14] and is the most widely used version of the Region-based CNN family. It uses a two-stage proposal and refinement of regions followed by classification. The model used in this pipeline was trained on 10,000 camera images from North East England, and the base model and training code are openly available [57,58]. YOLOv5 is the latest version of the YOLO series of models, released in May 2020 [59]. The YOLO architecture performs detection in a single pass with features extracted from cells of an image split into a grid feeding into a regression algorithm predicting objects’ bounding boxes. YOLOv5 provides different pre-trained models to balance speed and accuracy. In our experiments, we chose the YOLOv5s and YOLOv5x, where YOLOv5s is the lightest model with the fastest speed but the least accuracy, whilst YOLOv5x is the heaviest model with the slowest speed but the most accurate results. All the models were run on a Google cloud virtual machine using NVIDIA GPU Tesla T4 [60]. Table 4 shows a comparison of processing speed using the above models.

**Figure 11:**
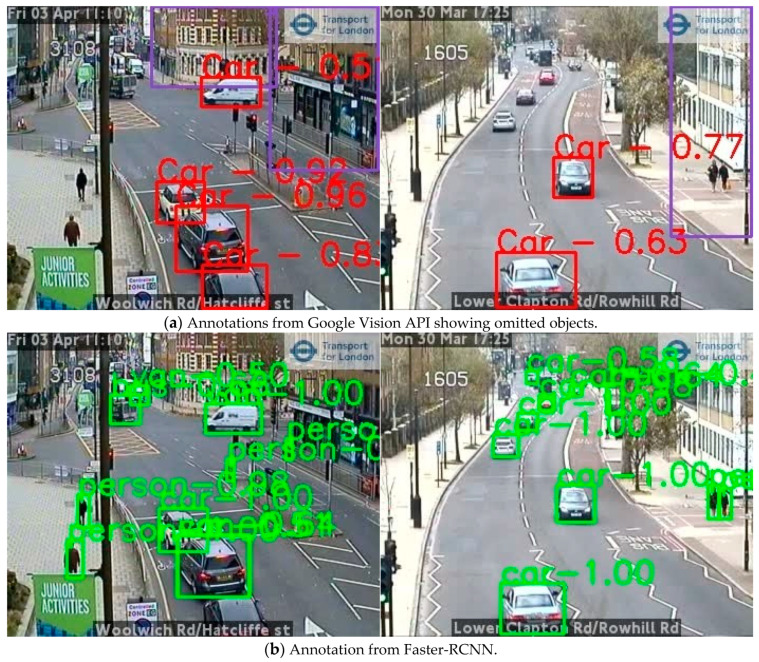

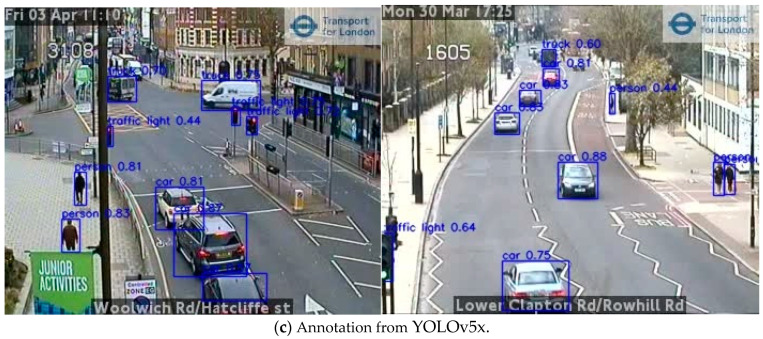
Comparison of automatic annotation results from different object detection models using two example images from London (images TfL Open Data). (**a**) Annotation from Google Vision API, (**b**) annotation from Faster-RCNN, (**c**) annotation from YOLOv5x. Numbers are the confidence scores associated with detected objects.

Figure 11 shows some example results from different models where the Google Vision API missed a few objects whilst both YOLOv5x and Faster-RCNN perform better with fewer omissions with differing confidence scores.

**Figure 12:**
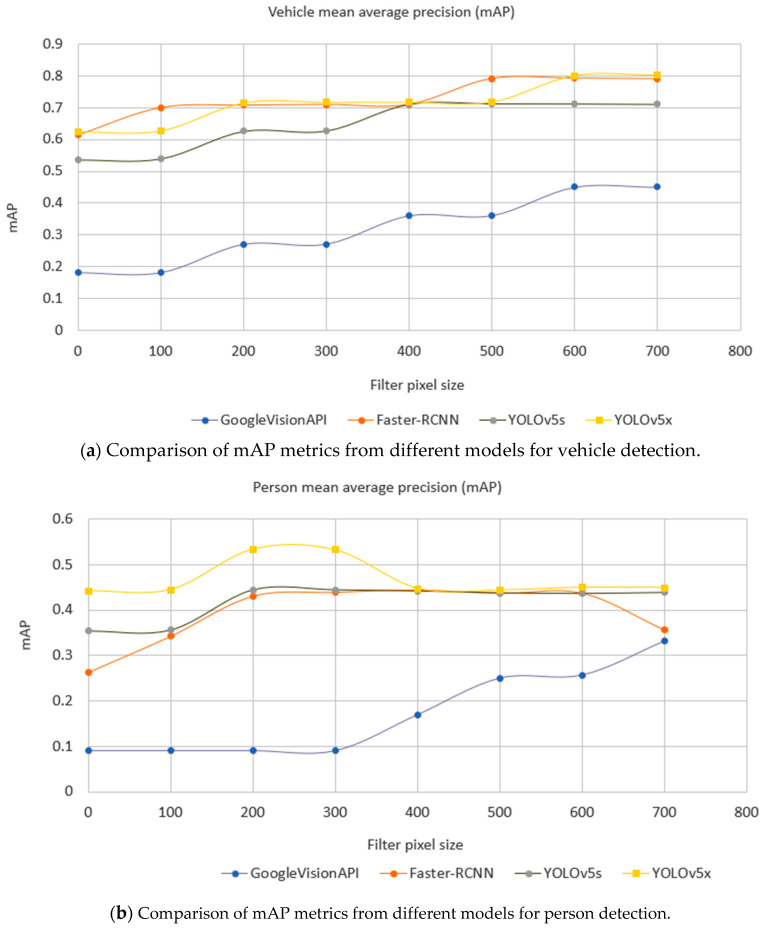
Comparison of mAP metric for different object detection models by minimum label size in pixels (horizontal axis) and label class (chart—vehicles top, people below), where the filter pixel size is zero. All labeled objects are considered.

A detailed comparison of performance quality is shown in Figure 12. This is based on an evaluation dataset of 1700 images captured from JamCam, London during the daytime detailed in Table 5. As the Google Vision API does not identify any cyclists and YOLOv5 does not provide a category of cyclist or motorcyclist, these classes have been omitted in the comparison. The performance of object detection is normally evaluated using mean Average Precision (mAP) over categories [61]. Both YOLOv5 and the Faster-RCNN achieved better mAP than the Google Vision API did.

Here, 1700 traffic camera images across London (see Table 5) were used to evaluate all the models. As it is important that the model is able to classify objects at different sizes, in relation to the distance a detected object is from the camera in a scene, we calculated the metrics to also consider an object size. This is shown by the horizontal axis, filter pixel size, in Figure 12. Here, zero means that the testing set includes all sizes of objects; 100 means that the testing set excludes any labeled objects smaller than 100 pixels and so on. Figure 12 shows that the bigger the object size, the better the performance. Importantly, it also shows there was a turning point of mAP against object sizes where a model becomes more stable, and this helps guide the camera settings. For example, in Figure 12a, Faster-RCNN became stable when the filter pixel size of vehicles was 500 pixels, while YOLOv5s became stable at 400 pixels. Faster-RCNN achieved the best performance amongst individual models in the category of vehicle, while YOLOv5x achieved the best against other individual models in the category of a person.

Another important factor is the cost. Google Vision API is billed per call [62]. The cost of using Faster-RCNN to create the weekly time series was about GBP 10 per day, whilst the Google Vision API charges about GBP 120 per day for the weekly time series. The YOLOv5 costs were similar to Faster-RCNN.

The Faster-RCNN and YOLO series have the code repositories on GitHub and this gives users flexibility to tailor the models on their targeted semantic objects or improve the accuracy through fine-tuning or re-training. However, the deployment and implementation of the Google Vision API is hidden from the end users.

#### 4.2.2. Investigation of Ensemble of Models

**Figure 13:**
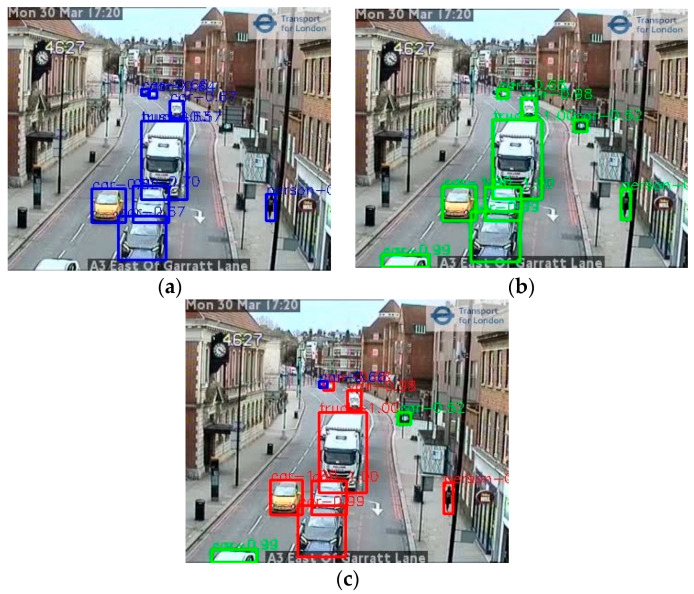
Example of object detection using an ensemble method; (**a**,**b**) Annotations detected by the Faster-RCNN and YOLOv5x models independently, respectively; (**c**) the result of combining the results in an ensemble. In (**c**), red boxes show objects detected by both models; green boxes are objects detected by Faster-RCNN but not YOLOv5x, and blue boxes are objects detected by YOLOv5x but not Faster-RCNN. (image TfL Open Data).

The above accuracy comparison indicates that the YOLOv5 and the Faster-RCNN models appeared to have particular strengths for different object detection types. Figure 13 shows an example.

To exploit these differences and improve accuracy, an ensemble method based on the Faster-RCNN and YOLOv5x models was designed. Ideally, a pool with three or more models can lead to an easy ensemble solution, such as a voting scheme, when conflicts arise from different models. In this case, only two models were available, so this presented a challenge to solve conflicts between two models. Through analyzing these two models’ behavior, we proposed a ‘max’ method that gives priority to the individual results with the maximum confidence scores from different models.

**Figure 14:**
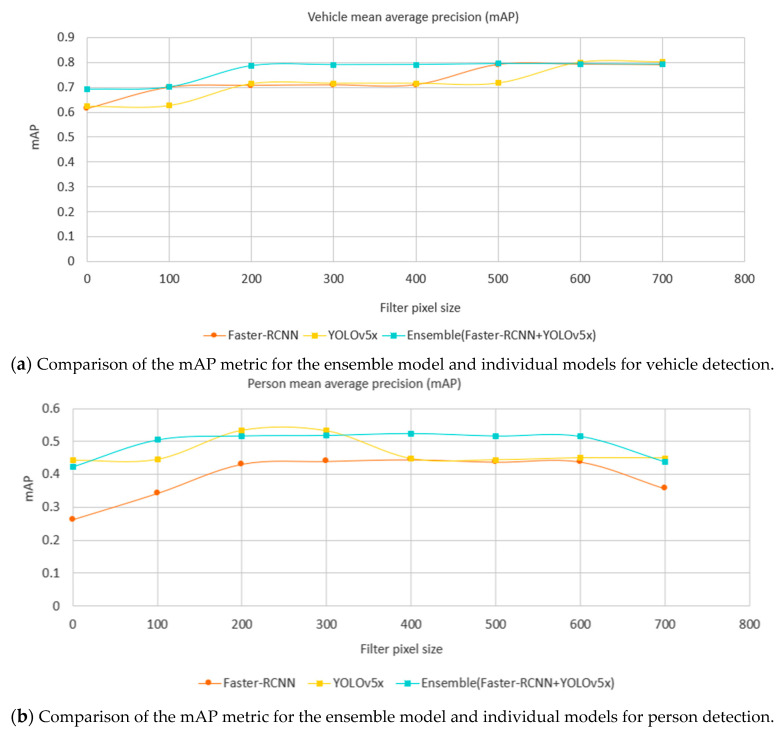
Comparison of the mAP metric for the ensemble model, and components of Faster-RCNN and YOLOv5x models by minimum label size in pixels (horizontal axis) and label class (chart; above vehicles, below people) showing the superior performance of the ensemble method over its constituents.

Recall the comparison of individual models in Figure 12 where Faster-RCNN achieved the best mAP on the category of vehicle, while YOLOv5x achieved the best on the category of person. Using the same evaluation dataset (Table 5), a comparison of the ensemble method against Faster-RCNN and YOLOv5x is shown in Figure 14 where the ensemble method achieves a better mAP than the individual models in general in both categories of vehicle and person. The ensemble method can be parallelized and the speed is limited by the slowest model in the pool.

### 4.3. Static Mask

The object detection process identifies both static and moving objects. As the aim is to detect activity, it is important to filter out static objects using temporal information from successive images as a post-processing operation. The images are usually sampled at 10 min intervals, but cameras may change angles irregularly, so traditional methods for background modeling in continuous video, such as mixture of Gaussians [63], were not suitable for such a long interval and fast camera view switches. The traditional background modeling normally requires a period of continuing video with a fixed view to build up a background image.

A Structure SImilarity Measure (SSIM) [64] approach was adopted to extract the background. SSIM is a perception-based model that perceives changes in structural information. Structural information is the idea that the pixels have strong interdependencies especially when they are spatially close. These dependencies carry important information about the structure of the objects in the visual scene. SSIM is not suitable to build up background/foreground [65] with continuous video, because the changes occur between two sequential frames over a very short period, e.g., 40 ms for 25 frames per second video, and so are too subtle. For a 10 min interval typical of traffic camera images though, SSIM has enough information to perceive visual changes within structural patterns.

**Figure 15:**
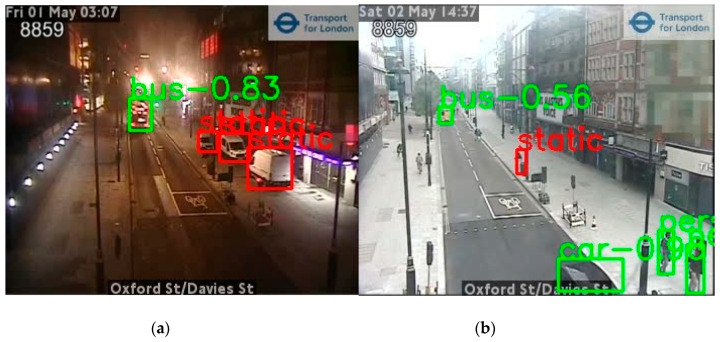
Examples of annotation results where a static mask has been applied in post-processing. (**a**,**b**) were taken from a camera from Oxford Street, London at different time where green boxes are moving objects and red boxes are static objects (images supplied by TfL Open Data).

Any pedestrians and vehicles classified during object detection were set as static and removed from the final counts if they also appeared in the background (Figure 15). Figure 15a illustrates an example where parked cars have been identified as static. In Figure 15b, a rubbish bin was misclassified as a pedestrian by the object detection but was filtered out as a static background element.

## 5. Statistical Processing

### 5.1. Imputation

Once the counts of objects were produced, a number of subsequent processing steps were carried out to: (a) compensate for missing or low-quality images, (b) aggregate the cameras to a town or city level, and (c) remove seasonality to facilitate interpretation of the results. These processes are necessary to make the time series comparable over time and to ensure that technical faults, such as camera downtime due to maintenance or nonfunctionality, are not counted as actual drops in activity.

The imputation consisted of identifying missing periods of data from each of the cameras (usually missing due to technical faults), and then replacing the missing counts of each object type by estimated values. This ensures that missing images or outages in the original data do not lead to artificial drops in the estimated level of the time series. The imputation was carried out using seasonal decomposition. First, the seasonality of the series was removed from the series. Secondly, the mean of the series within a specified time window was imputed to the missing periods. Finally, the seasonality was reapplied to the imputed series. This results in a time series that displays the same seasonality in the missing periods as the original series. The imputation was applied using the seasonally decomposed missing value imputation method (seadec) from the ImputeTS package in R [66], and was found to be the most suitable imputation method out of those assessed. Other methods such as imputation by interpolation, the last observation carried forward, and Kalman Smoothing and State Space Models were considered; however, these yielded less satisfactory results and, in the case of imputation by Kalman Smoothing, the compute time was significantly longer and unfeasible for production systems.

Following imputation, the counts of each object type were aggregated to each of the locations of interest. For example, all the cars counted in the city of Reading by the traffic cameras (including imputed counts) were summed to produce the corresponding daily time series for Reading.

### 5.2. Seasonal Adjustment

Once the series had been aggregated, they were seasonally adjusted to make the interpretation of the results clearer. Seasonal adjustment removes fluctuations due to the time of day or day of the week from the data, making it easier to observe underlying trends in the data. Given that these are higher-frequency time series relative to those traditionally used in official statistics, we applied seasonal adjustment using the TRAMO/SEATS method in JDemetra+, as implemented in the R JDemetra High Frequency package (rjdhf) [67]. The adjustment was applied on both the hourly and daily time series. The presence of different types of outliers was tested during seasonal adjustment to ensure that these do not distort the estimation when it is decomposed into trend, seasonal, and irregular components. Seasonally adjusting the data led to the possibility of achieving negative values for some time series in cases in which the seasonal component was negative and the observed count was very low (e.g., zero). This is a problem often found in time series with strong seasonal movements and, as traffic counts are a nonnegative time series, a transformation was required. A square root transformation was applied to the data, prior to seasonal adjustment, in order to remove the possibility of artificially creating negative counts.

## 6. Validation of Time Series

### 6.1. Time Series Validation against ANPR

Although mean average precision and precision-recall measure the models’ performance, it was important to check the consistency of time series data produced against other sources. Traffic counts from Automatic Number Plate Recognition (ANPR) data obtained from Newcastle University’s Urban Observatory project and Tyne and Wear UTMC were used as ground truths to compare against the raw time series generated from the machine learning pipeline.

ANPR traffic counts are defined on road segments with a pair of cameras, where a vehicle count is registered if the same number plate is recorded at both ends of the segment. This has the effect of not including vehicles that turn off onto a different road before they reach the second camera; such vehicles would appear in traffic camera images and so counts may not be entirely consistent between the two sources if this happens often. To this end, we identified short ANPR segments that also contained traffic cameras. We used the Faster-RCNN model to generate traffic counts from these cameras and compared these against the ANPR data.

**Figure 16:**
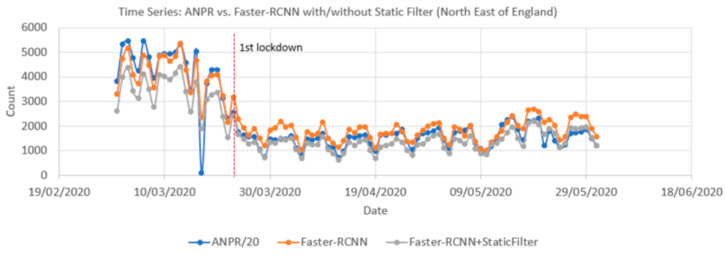
Comparison of traffic count time series produced from Automatic Number Plate Recognition (ANPR) and object detection using open traffic cameras (with and without a filter for static objects) for the same locations between 1 March 2020 and 31 May 2020. The date of the first UK Covid-19 lockdown is also shown.

Figure 16 compares time series from ANPR data and the Faster-RCNN model (with and without the static mask) from 1 March to 31 May 2020. Pearson correlation coefficients showed a significant positive correlation between the raw time series and the ANPR data as high as 0.96. The vehicle count from the pipeline is only calculated every 10 min, whilst ANPR is continuous, so to harmonize the rate at which the two methods capture vehicles, the ANPR count was downscaled by a factor of 20. This assumes a camera image covers a stretch of road that would take 30 s for a car to travel along. As the camera only samples every 10 min (600 s), we assume we have captured 30 s out of the 600 s the ANPR will have recorded, i.e., 1/20th.

**Figure 17:**
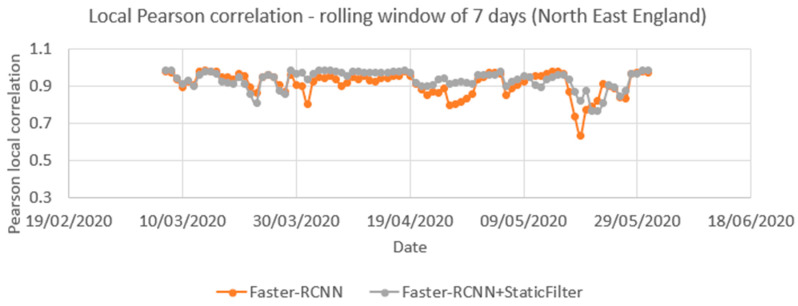
Correlation between time series produced from Automatic Number Plate Recognition (ANPR) and object detection using open traffic camera (with and without a filter for static objects) for the same locations between 1 March 2020 and 31 May 2020) based on a local Pearson correlation coefficient that uses a rolling seven-day window.

Figure 17 compares the local correlation coefficients for the time series over a rolling seven-day window. The series is nearly the same for the period of 1 March and 31 May with or without the static mask, though that with the static mask shows less variation. The average of these local Pearson correlations was 0.91 for Faster-RCNN and 0.93 for the Faster-RCNN+static mask.

### 6.2. Comparison with Department for Transport Data

**Figure 18:**
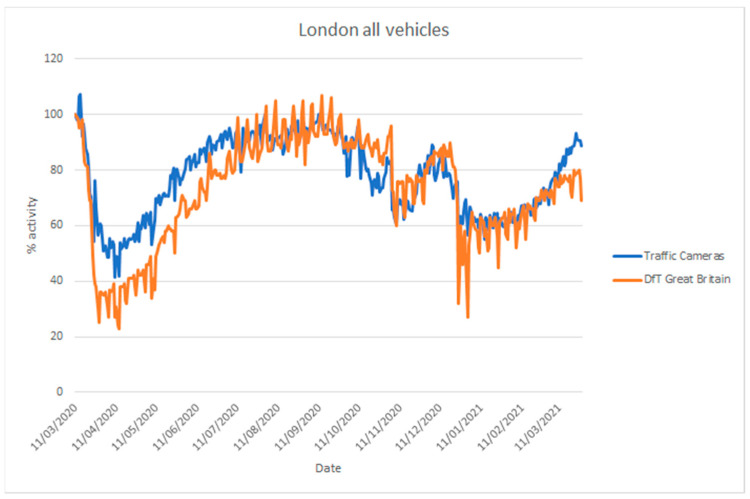
Comparison of time series describing traffic levels between 11 March 2020 and 28 March 2021 based on road traffic statistics for Great Britain produced by the Department for Transport (DfT) using automatic traffic counters and traffic cameras from London (TfL). Traffic levels are given as a percentage activity index baselined to counts, from the respective source, on 11 March 2020.

**Figure 19:**
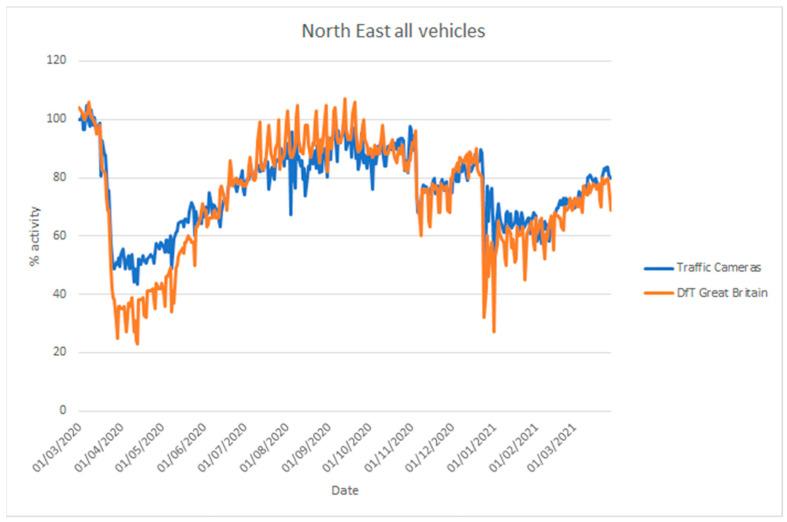
Comparison of time series describing traffic levels between 1 March 2020 and 28 March 2021 based on road traffic statistics for Great Britain produced by the Department for Transport (DfT) using automatic traffic counters and data produced by traffic cameras from North East England (NE travel data). Traffic levels are given as a percentage activity index baselined to counts, from the respective source, on 11 March 2020.

The UK Department for Transport (DfT) produces statistics on road traffic aggregated for Great Britain from 275 automatic traffic count sites [68]. In addition, they provide statistics on cyclists, bus travel, and rail travel. Their vehicle counts are disaggregated into cars, light commercial vehicles, and heavy goods vehicles. These data are not directly comparable to the traffic camera data [5], as the coverage is different (Great Britain-wide as opposed to a selection of towns and cities), but they are useful to assess whether the traffic camera sources can be used to estimate inflection points in traffic. Figure 18 and Figure 19 compare vehicle activity from the traffic camera sources from London and North East England, respectively, with DfT traffic data for Great Britain [69].

Considering the differences in coverage, and the location of count sites (highways, urban, and rural) and traffic cameras (solely urban), both sources provide similar inflection points in activity coinciding with the introduction of lockdown measures in late March 2020, during the month of November 2020 and from late December in 2020. Similarly, both sources provide a similar trend of increasing activity when restrictions are gradually lifted, with somewhat differing levels. The traffic cameras we applied cover different areas from DfT data and, moreover, they cover pedestrian counts in commercial or residential areas that the DfT data do not cover.

## 7. Discussion

Traffic camera data can offer tremendous value to public authorities by providing real-time statistics to monitor levels of activity in local populations. This can inform local and national policy interventions such as those seen during the COVID-19 pandemic, especially where regional and local trends may differ widely. This type of traffic camera analysis is best suited at detecting trends in ‘busyness’ or activity in town and city centers, but is not suited for estimating the overall amount of vehicle or pedestrian movements, or for understanding the journeys that are made given the periodic nature of the images provided. It provides high frequency (hourly) and timely (weekly) data, which can help to detect trends and inflection points in social behavior. As such, it provides local insights that complement other mobility, and supplement other transport, datasets that lack such local and temporal granularity. For example, Google’s mobility data [70] are aggregated from Android users who have turned on location history on their smartphones but aggregated by region; Highways England covers motorways and some A-roads; commercial mobile photo cell data are not widely available on a continuous basis and can be expensive. These openly accessible traffic cameras are mainly focused on junctions, and residential and commercial areas, and thereby capture a large proportion of vehicles and pedestrians regardless of journey purpose in near real-time. Moreover, as these sensors are already in place and images are relatively accessible, it provides a scalable and efficient means of garnering statistics.

Realizing the value of the data within these images requires the application of data science, statistical, and data engineering techniques that can produce robust results despite the wide variability in quality and characteristics of the input camera images. Here, we have demonstrated methods that are reliable in achieving a reasonable degree of accuracy and coherence with other sources. However, despite the success of the developed pipeline in providing operational data and as a beacon for future development, there remains a number of challenges to address. Although we have demonstrated outputs from a range of locations across the UK, the coverage of open cameras is not comprehensive, and so the selection has been led by availability and ease of access. Within locations, cameras are sited principally to capture information about traffic congestion; hence, places without cars such as pedestrianized shopping districts are entirely absent in some areas. Validation of the data was successful but reliable metrics on accuracy are problematic as the accuracy of detecting different object types depends on many factors such as occlusion of objects, weather, illumination, choice of machine learning models, training sets, and camera quality and setting. Some factors such as camera settings are also outside of our immediate control. Current social distancing has meant that the density of pedestrians observed is sufficiently low for them to be satisfactorily detected. However, we would not expect the current models to perform so well for very crowded pavements as levels of occlusion, etc. would increase. The camera images are typically produced every 10 min, which limits the scope of the insights that can be produced and, therefore, its use for the management of flows should not be overstated. The data nonetheless have real value in capturing, describing, and expressing the relative change in the busyness in our cities and, therefore, the effectiveness of some policy interventions.

## 8. Conclusions

Using publicly available traffic cameras as a source of data to understand changing levels of activity within society offers many benefits. The data are very timely with images being made available in real-time. While we have demonstrated the processing of these in batch mode, there are no technical barriers to more frequent, near real-time updates. Data outputs can also easily be scaled to hourly, daily, and weekly summaries.

The camera as a sensor enables a large array of different objects to be detected: cars, buses, motorcycles, vans, pedestrians, etc. Additional objects, such as e-scooters and e-bikes, can be added to the models with further training. These camera systems already exist widely and reusing and repurposing them make further use of existing public resources. There is no additional cost for the deployment and upkeep of expensive infrastructure as their maintenance and upgrades are managed as part of existing agreements for the primary purpose of traffic management and/or public safety. The additional cost for processing this data is also relatively modest for our architecture less than GBP 1000 (USD 1200) per month, which is significantly less than the cost of a single traffic counting device such as a radar-based device.

Whilst not always currently open, camera coverage of town and city centers, which is the locus of much economic activity, is generally comprehensive, and most cities, and many smaller towns, already have cameras in place that can readily provide information on commuting, retail, and pedestrian flows. It is feasible to carry this out on a national scale with respect to cost, coverage, and digital infrastructure. There are particular benefits of providing regional updates to national government when there are distinct differences in policies (e.g., localized lockdown regimes).

The data used here are in the public domain and uses low-resolution imagery from which it is difficult or impossible to personally identify individuals. The use of time-separated still images means that tracking or other privacy-invasive practices are largely impossible. The processing pipeline design ensures that summaries and counts are the only things that are stored, and images, once processed, can be deleted. As these cameras provide data in near real-time, there is also the potential for low-latency applications with data provided within minutes of upload.

The further uptake of this approach and benefits could be realized if more local authorities and organizations made their traffic camera imagery data available on a low-resolution, infrequent, and open licensing basis; non-highways coverage would be especially helpful and could also help people to avoid crowding and queues at retail sites. Standardization in the way imagery is exposed would reduce the complexity of scaling the approach nationally or internationally and could ensure there is consistency in the data frequency and aggregation. Commercial pedestrian and vehicle counting systems are also available for CCTV, but additional transparency around the algorithms used and their efficacy is desirable before combining different approaches and processing pipelines.

As further work, we intend to make a number of technical enhancements, including improving the sampling design and scoring system for selecting cameras and investigating methods for analyzing crowds in images. We will also look at how the data, and related social and environmental factors, might predict activity at future time points, in locations without cameras, or in response to proposed public policy interventions.

## Figures and Tables

**Table 1:** Sources of open traffic camera image data used in this research and date when ingestion of data from the source was commenced.

Image Source	Date First Ingested
Durham	7 May 2020
Transport for London (TfL)	11 March 2020
Transport for Greater Manchester (TfGM)	17 April 2020
North East England (NE Travel Data)	1 March 2020
Northern Ireland	15 May 2020
Southend	7 May 2020
Reading	7 May 2020

**Table 2:** Scores given for the presence of different types of scene elements within the view of a traffic camera, used to assess the camera’s potential to describe the ‘busyness’ of the local environment.

Object	Score
Shops	+5
Residential	+5
Pavement	+3
Cycle lane	+3
Bus lane	+1
Traffic lights	+1
Roundabout	−3

**Table 3:** Counts of labels generated from two collections of image data (London and North East England) by type of object annotated.

	Images	Car	Van	Truck	Bus	Pedestrian	Cyclist	Motorcyclist
**London**	1950	9278	1814	1124	1098	3083	311	243
**North East England**	2104	9968	1235	186	1699	2667	57	43
**Total**	4054	19,246	3049	1310	2797	5750	368	286

**Table 4:** Comparison of average processing speeds (milliseconds per image) measured for different object detection models.

	Google Vision API	Faster-RCNN	YOLOv5s	YOLOv5x
**Speed** (ms/image)	109	35	14	31

**Table 5:** Number of objects of different categories collected in the evaluation dataset.

Object Class	Count of Objects
Car	8570
Bus	958
Truck	1044
Van	1593
Person	2879
Cyclist	281
Motorcyclist	219

## Data Availability

The code associated with this paper is available at https://github.com/datasciencecampus/chrono_lens (accessed on 28 June 2021).

## Appendix A

**Table A1:** Publicly available camera sources identified as part of this research. The links are accessed on 28 June 2021.

Location	Link
Aberdeen	https://online.aberdeencity.gov.uk/services/Webcam/Default.aspx
Andover	https://www.romanse.org.uk/CCTVAndover.htm
Bude	https://www.budewebcam.co.uk/#
Cinderford	https://www.cinderfordtowncouncil.gov.uk/webcam/
Dublin	https://www.dublincity.ie/dublintraffic/
Durham	https://www.durham.gov.uk/durhamtrafficcameras
Farnham	http://www.camsecure.co.uk/farnham_webcam.html
Glasgow	https://glasgow.gov.uk/webcam
Kent	https://www.kenttraffic.info
Leeds	http://www.leedstravel.info/cdmf-webserver/jsp/livecctv.jsp
London (TfL)	https://www.tfljamcams.net
Manchester (TfGM)	https://tfgm.com/roads/live-traffic-cameras
North East England	https://www.netrafficcams.co.uk/map https://www.netraveldata.co.uk/
Northern Ireland	https://trafficwatchni.com/twni/cameras
Nottingham	https://www.itsnottingham.info
Reading	http://www.reading-travelinfo.co.uk/live-traffic-cameras.aspx
Sheffield	https://www.sheffield.gov.uk/trafficcameras#
Shropshire	https://www.shropshire.gov.uk/webcams/views-from-theatre-severn/
Southampton	https://romanse.org.uk/CCTVSoton.htm
Southend	https://sbctraffic.southend.gov.uk
Sunderland	https://www.sunderland.gov.uk/article/16148/Webcams-
Wrexham	https://beta.wrexham.gov.uk/service/wrexham-webcams/live-webcam-llwyn-isaf

## References

[1] Feng W., Ji D., Wang Y., Chang S., Ren H., Gan W. (2018). Challenges on large scale surveillance video analysis. 2018 IEEE/CVF Conference on Computer Vision and Pattern Recognition Workshops (CVPRW).

[2] Liu X., Liu W., Ma H., Fu H. (2016). Large-scale vehicle re-identification in urban surveillance videos. 2016 IEEE International Conference on Multimedia and Expo (ICME).

[3] Sochor J., Spanhel J., Herout A. (2019). BoxCars: Improving fine-grained recognition of vehicles using 3-D bounding boxes in traffic surveillance. IEEE Trans. Intell. Transp. Syst..

[4] Spanhel J., Sochor J., Makarov A. (2018). Vehicle fine-grained recognition based on convolutional neural networks for real-world applications. 2018 14th Symposium on Neural Networks and Applications (NEUREL).

[5] Coronavirus and the Latest Indicators for the UK Economy and Society Statistical Bulletins. https://www.ons.gov.uk/peoplepopulationandcommunity/healthandsocialcare/conditionsanddiseases/bulletins/coronavirustheukeconomyandsocietyfasterindicators/previousReleases.

[6] Buch N., Velastin S.A., Orwell J. (2011). A review of computer vision techniques for the analysis of urban traffic. IEEE Trans. Intell. Transp. Syst..

[7] Bautista C.M., Dy C.A., Mañalac M.I., Orbe R.A., Ii M.C. (2016). Convolutional neural network for vehicle detection in low resolution traffic videos. IEEE Region 10 Symposium (TENSYMP).

[8] Zhang S., Benenson R., Schiele B. CityPersons: A diverse dataset for pedestrian detection. Proceedings of the 2017 IEEE Conference on Computer Vision and Pattern Recognition (CVPR).

[9] Farias I.S., Fernandes B.J.T., Albuquerque E.Q., Leite B.L.D. Tracking and counting of vehicles for flow analysis from urban traffic videos. Proceedings of the 2017 IEEE Latin American Conference on Computational Intelligence (LA-CCI).

[10] Idé T., Katsuki T., Morimura T., Morris R. (2017). City-wide traffic flow estimation from a limited number of low-quality cameras. IEEE Trans. Intell. Transp. Syst..

[11] Fedorov A., Nikolskaia K., Ivanov S., Shepelev V., Minbaleev A. (2019). Traffic flow estimation with data from a video surveillance camera. J. Big Data.

[12] Redmon J., Divvala S., Girshick R., Farhadi A. You only look once: Unified, real-time object detection. Proceedings of the 2016 IEEE Conference on Computer Vision and Pattern Recognition (CVPR).

[13] Espinosa J.E., Velastin S.A., Branch J.W. (2017). Vehicle detection using alex net and faster R-CNN deep learning models: A comparative study. Advances in Visual Informatics (IVIC).

[14] Ren S., He K., Girshick R., Sun J. (2017). Faster R-CNN: Towards real-time object detection with region proposal networks. IEEE Trans. Pattern Anal. Mach. Intell..

[15] Wen X., Shao L., Xue Y., Fang W. (2015). A rapid learning algorithm for vehicle classification. Inf. Sci..

[16] Feris R.S., Siddiquie B., Petterson J., Zhai Y., Datta A., Brown L.M., Pankanti S. (2012). Large-scale vehicle detection, indexing, and search in urban surveillance videos. IEEE Trans. Multimed..

[17] Fraser M., Elgamal A., He X., Conte J.P. (2010). Sensor network for structural health monitoring of a highway bridge. J. Comput. Civ. Eng..

[18] Semertzidis T., Dimitropoulos K., Koutsia A., Grammalidis N. (2010). Video sensor network for real-time traffic monitoring and surveillance. IET Intell. Transp. Syst..

[19] Rezazadeh Azar E., McCabe B. (2012). Automated visual recognition of dump trucks in construction videos. J. Comput. Civ. Eng..

[20] Chen Y., Wu B., Huang H., Fan C. (2011). A real-time vision system for nighttime vehicle detection and traffic surveillance. IEEE Trans. Ind. Electron..

[21] Shi H., Wang Z., Zhang Y., Wang X., Huang T. Geometry-aware traffic flow analysis by detection and tracking. Proceedings of the 2018 IEEE/CVF Conference on Computer Vision and Pattern Recognition Workshops (CVPRW), Salt Lake City.

[22] Kumar A., Khorramshahi P., Lin W., Dhar P., Chen J., Chellappa R. A Semi-automatic 2D solution for vehicle speed estimation from monocular videos. Proceedings of the 2018 IEEE/CVF Conference on Computer Vision and Pattern Recognition Workshops (CVPRW), Salt Lake City.

[23] Hua S., Kapoor M., Anastasiu D.C. Vehicle tracking and speed estimation from traffic videos. Proceedings of the 2018 IEEE/CVF Conference on Computer Vision and Pattern Recognition Workshops (CVPRW).

[24] Tran M., Dinh-duy T., Truong T., Ton-that V., Do T., Luong Q.-A., Nguyen T.-A., Nguyen V.-T., Do M.N. Traffic flow analysis with multiple adaptive vehicle detectors and velocity estimation with landmark-based scanlines. Proceedings of the 2018 IEEE/CVF Conference on Computer Vision and Pattern Recognition Workshops (CVPRW), Salt Lake City.

[25] Huang T. Traffic speed estimation from surveillance video data. Proceedings of the 2018 IEEE/CVF Conference on Computer Vision and Pattern Recognition Workshops (CVPRW), Salt Lake City.

[26] Fan Q., Brown L., Smith J. (2016). A closer look at Faster R-CNN for vehicle detection. 2016 IEEE Intelligent Vehicles Symposium (IV).

[27] Project Odysseus–Understanding London ‘Busyness’ and Exiting Lockdown. https://www.turing.ac.uk/research/research-projects/project-odysseus-understanding-london-busyness-and-exiting-lockdown.

[28] Quantifying Traffic Dynamics to Better Estimate and Reduce Air Pollution Exposure in London. https://www.dssgfellowship.org/project/reduce-air-pollution-london/.

[29] James P., Das R., Jalosinska A., Smith L. (2020). Smart cities and a data-driven response to COVID-19. Dialogues Hum. Geogr..

[30] Peppa M.V., Bell D., Komar T., Xiao W. (2018). Urban traffic flow analysis based on deep learning car detection from CCTV image series. ISPRS–Int. Arch. Photogramm. Remote Sens. Spat. Inf. Sci..

[31] Uo-Object_Counting API. https://github.com/TomKomar/uo-object_counting.

[32] Using Spare CCTV Capacity to Monitor Activity Levels during the COVID-19 Pandemic. http://www.ubdc.ac.uk/news-media/2020/april/using-spare-cctv-capacity-to-monitor-activity-levels-during-the-covid-19-pandemic/.

[33] Creating Open Data Counts of Pedestrians and Vehicles using CCTV Cameras. http://www.ubdc.ac.uk/news-media/2020/july/creating-open-data-counts-of-pedestrians-and-vehicles-using-cctv-cameras/.

[34] Vivacity: The 2m Rule: Are People Complying?. https://vivacitylabs.com/2m-rule-are-people-complying/.

[35] Coronavirus and the Latest Indicators for the UK Economy and Society. https://www.spring-board.info/news-media/news-post/coronavirus-and-the-latest-indicators-for-the-uk-economy-and-society.

[36] Aloi A., Alonso B., Benavente J., Cordera R., Echániz E., González F., Ladisa C., Lezama-Romanelli R., López-Parra Á., Mazzei V. (2020). Effects of the COVID-19 lockdown on urban mobility: Empirical evidence from the city of Santander (Spain). Sustainability.

[37] Costa C., Chatzimilioudis G., Zeinalipour-Yazti D., Mokbel M.F. (2017). Towards real-time road traffic analytics using telco big data. Proceedings of the International Workshop on Real-Time Business Intelligence and Analytics.

[38] Zambrano-Martinez J., Calafate C., Soler D., Cano J.-C., Manzoni P. (2020). Modeling and characterization of traffic flows in urban environments. Sensors.

[39] Naphade M., Anastasiu D.C., Sharma A., Jagarlamudi V., Jeon H., Liu K., Chang M.-C., Lyu S., Gao Z. (2017). The NVIDIA AI City Challenge. 2017 IEEE SmartWorld, Ubiquitous Intelligence & Computing, Advanced & Trusted Computed, Scalable Computing & Communications, Cloud & Big Data Computing, Internet of People and Smart City Innovation (SmartWorld/SCALCOM/UIC/ATC/CBDCom/IOP/SCI).

[40] Naphade M., Wang S., Anastasiu D.C., Tang Z., Chang M.-C., Yang X., Zheng L., Sharma A., Chellappa R., Chakraborty P. The 4th AI city challenge. Proceedings of the 2020 IEEE/CVF Conference on Computer Vision and Pattern Recognition Workshops (CVPRW).

[41] Wen L., Du D., Cai Z., Lei Z., Chang M.-C., Qi H., Lim J., Yang M.-H., Lyu S. (2020). UA-DETRAC: A new benchmark and protocol for multi-object detection and tracking. Comput. Vis. Image Underst..

[42] Tang Z., Naphade M., Liu M.-Y., Yang X., Birchfield S., Wang S., Kumar R., Anastasiu D.C., Hwang J.-N. CityFlow: A city-scale benchmark for multi-target multi-camera vehicle tracking and Re-identification. Proceedings of the 2019 IEEE/CVF Conference on Computer Vision and Pattern Recognition (CVPR).

[43] Shao S., Zhao Z., Li B., Xiao T., Yu G., Zhang X., Sun J. CrowdHuman: A Benchmark for Detecting Human in a Crowd. https://arxiv.org/abs/1805.00123.

[44] Leal-Taixé L., Milan A., Reid I., Roth S., Schindler K. (2015). MOTChallenge 2015: Towards a Benchmark for Multi-Target Tracking. https://arxiv.org/abs/1504.01942.

[45] Cordts M., Omran M., Ramos S., Rehfeld T., Enzweiler M., Benenson R., Franke U., Roth S., Schiele B. (2016). The Cityscapes Dataset for Semantic Urban Scene Understanding. https://arxiv.org/abs/1604.01685.

[46] Zhang S., Wu G., Costeira J.P., Moura J.M.F. FCN-rLSTM: Deep spatio-temporal neural networks for vehicle counting in city cameras. Proceedings of the 2017 IEEE International Conference on Computer Vision (ICCV).

[47] Dollar P., Wojek C., Schiele B., Perona P. (2012). Pedestrian detection: An evaluation of the state of the art. IEEE Trans. Pattern Anal. Mach. Intell..

[48] Braun M., Krebs S., Flohr F., Gavrila D.M. (2019). EuroCity persons: A novel benchmark for person detection in traffic scenes. IEEE Trans. Pattern Anal. Mach. Intell..

[49] Geiger A., Lenz P., Stiller U.R. (2013). Vision meets robotics: The KITTI dataset. Int. J. Robot. Res..

[50] Urban Observatory–Camera Views. https://api.newcastle.urbanobservatory.ac.uk/camera/.

[51] United Nations Global Platform: Hosted Instance of VGG Image Annotator. https://annotate.officialstatistics.org/.

[52] VGG Image Annotator (VIA). https://www.robots.ox.ac.uk/~vgg/software/via/.

[53] United Nations Global Platform. https://marketplace.officialstatistics.org/.

[54] Google BigQuery. https://cloud.google.com/bigquery.

[55] Google Cloud Functions. https://cloud.google.com/functions.

[56] Google Vision API. https://cloud.google.com/vision.

[57] TensorFlow 1 Detection Model Zoo. https://github.com/tensorflow/models/blob/master/research/object_detection/g3doc/tf1_detection_zoo.md.

[58] Training and Evaluation with TensorFlow 1. https://github.com/tensorflow/models/blob/master/research/object_detection/g3doc/tf1_training_and_evaluation.md.

[59] YOLOv5 in PyTorch. https://github.com/ultralytics/yolov5.

[60] Google GPUs on Compute Engine. https://cloud.google.com/compute/docs/gpus.

[61] Everingham M., Van Gool L., Williams C.K.I., Winn J., Zisserman A. (2010). The Pascal Visual Object Classes (VOC) challenge. Int. J. Comput. Vis..

[62] Google Pricing. https://cloud.google.com/vision/pricing.

[63] Xu Y., Dong J., Zhang B., Xu D. (2016). Background modeling methods in video analysis: A review and comparative evaluation. CAAI Trans. Intell. Technol..

[64] Wang Z., Bovik A.C., Sheikh H.R., Simoncelli E.P. (2004). Image quality assessment: From error visibility to structural similarity. IEEE Trans. Image Process..

[65] Bouwmans T., Silva C., Marghes C., Zitouni M.S., Bhaskar H., Frelicot C. (2018). On the role and the importance of features for background modeling and foreground detection. Comput. Sci. Rev..

[66] Moritza S., Bartz-Beielstein T. (2017). imputeTS: Time Series Missing Value Imputation in R. R J..

[67] Rjdhighfreq. https://github.com/palatej/rjdhighfreq.

[68] Transport Use during the Coronavirus (COVID-19) Pandemic. https://www.gov.uk/government/statistics/transport-use-during-the-coronavirus-covid-19-pandemic.

[69] Department for Transport: Road Traffic Statistics. https://roadtraffic.dft.gov.uk/.

[70] Google COVID-19 Community Mobility Reports. https://www.google.com/covid19/mobility/.

